# Why are long-lived plasma cells long-lived?

**DOI:** 10.3389/fimmu.2026.1785563

**Published:** 2026-05-07

**Authors:** Julia Grace Reinke, Christopher Schorr, Kelvin Paul Lee

**Affiliations:** 1 Department of Microbiology and Immunology, Indiana University, School of Medicine, Indianapolis, IN, United States; 2 Department of Biomedical Engineering, Purdue University, West Lafayette, IN, United States; 3 Department of Medicine, Indiana University, School of Medicine, Indianapolis, IN, United States; 4 Indiana University Simon Comprehensive Cancer Center, Indiana University, School of Medicine, Indianapolis, IN, United States

**Keywords:** humoral immunity, metabolism, multiple myeloma, niche, plasma cell, survival

## Abstract

Long-Lived Plasma Cells (LLPCs) are an integral part of long-term protective humoral immunity. They can live for decades, unlike Short-Lived Plasma Cells (SLPCs), and continuously produce antibody regardless of antigen stimulation, unlike Memory B Cells (MBCs). LLPCs are critical for the sustained protective immunity against pathogens that are only intermittently present in a population but can cause significant morbidity/mortality when active—such as epidemic diseases. What drives B cell differentiation specifically to the LLPC lineage is still not fully understood; there is conflicting information on what drives the fate decisions for MBCs vs. SLPCs vs. LLPCs. Evidence suggests that although LLPC and SLPC have similar gene transcriptional profiles they differ significantly in their metabolic profiles–likely due to the demands of prolonged continuous antibody production in LLPC. These metabolic changes include increased uptake of metabolic substrates, increased mitochondrial mass/function and enhanced fuel availability via lipophagy, and enhanced proteostasis to remove misfolded proteins. However, the possibility of repeated antigen-driven generation of a large number of highly metabolically active long-lived cells is problematic for a resource-constrained organism, and it is now clear that LLPC numbers are constrained by a limited number of specialized LLPC niches in the bone marrow and other tissues. LLPCs are not intrinsically long-lived but rely on interactions with the LLPC niche to maintain their longevity. For example, activation of the CD28 receptor on LLPC by its ligands CD80/CD86 on dendritic cells (DC) in the LLPC niche results in augmented metabolism through enhanced lipophagy, intracellular long chain fatty acid availability, oxidative phosphorylation, increased mitochondrial mass and function that are necessary for LLPC survival. CD28 activation is essential for the survival of LLPC but not for SLPC, supporting the concept that enhancement of LLPC metabolic capacity by interactions with its niche plays a key role in LLPC longevity. In human health, new insights into how LLPCs survive and differentiate will impact the development of robust and long-lasting vaccinations, as well as with treatment of autoantibody-mediate autoimmune diseases and PC malignancies such as multiple myeloma (MM)—as these malignancies remain dependent on many of the same survival pathways as their nonmalignant LLPC counterparts.

## 1 Identification of sustained protective humoral immunity and long-lived plasma cells

Disease is a major threat to the survival of all species, and for humanity the epidemic diseases have had (and continue to have) enormous impact on populations, societies and civilizations. Thus, sustained protective immunity against these diseases has been integral to human survival and evolution—and as such humans have been striving to understand it for millennia. The Athenian historian Thucydides wrote one of the earliest surviving observations of long-term immunity in the late 5^th^ century BCE. In his *History of the Peloponnesian War*, Thucydides noted that people who fell ill but survived the 430 BCE Plague of Athens would rarely fall ill with the same disease again, and if they did it was not fatal (1, 2). Long before the advent of germ theory people had noticed that prior exposure to a disease could protect against reoccurrence and they found ways to utilize that knowledge. There is evidence as early as 1000 CE that some areas of China, India, Turkey and Africa practiced early forms of inoculation against smallpox by exposing healthy individuals to the scabs or pus of the infected through cuts or inhalation (3–5). In 1796 Dr. Edward Jenner employed a similar method, using cowpox pus to protect against smallpox—coining the term *vaccination*. By the late 19^th^ century Dr. Louis Pasteur and his colleges had developed germ theory and with it new vaccines against anthrax and rabies, although how the body learned to protect itself against the diseases once exposed remained a mystery.

Modern understanding of humoral immunity began in 1890 when von Behring and Kitasato discovered that serum from rabbits infected with tetanus or diphtheria could protect mice from infection and used that antiserum to treat humans (6, 7). Immunologist Paul Ehrlich worked to discover the structure of antibodies through the early 1900’s, and in 1947 Dr. Astrid Fagraeus determined that a group of “Plasma Cells” (PCs), found in the red pulp of the spleen were responsible for antibody formation (8). Through the 20^th^ century scientists would discover different antibody types and begin to classify different types of antibody-producing cells, leading to considerable debate over which cell type was responsible for long-term humoral immunity and through what mechanisms (9).

For much of the mid 20^th^ century the scientific consensus was that all PC were short-lived, lasting only a few days or weeks, and that long-term humoral immunity was thought to come solely from reactivation of Memory B Cells (MBCs) upon antigen re-exposure (10). However, not all studies supported this, as Miller and Cole demonstrated in 1967 that a small population of PC were radiation resistant and could survive and produce antibodies for at least three months following irradiation (11). Similarly, Mattioli and Tomasi in 1973 reported that IgA-secreting PC could persist for 45 days in the intestine, and speculated that there were two populations of PC, one being longer-lived (12).

Further evidence for a distinct subset of PC that were long lived was reported by Manz and colleagues in 1997 showing that PC in the bone marrow had a lifespan of at least three months (13). Shortly afterwards, Slifka and colleagues found that LCMV-infected mice continued to produce high levels LCMV-specific IgG even after depletion of MBCs by irradiation – demonstrating that radiation-resistant PC were responsible for durable long-term humoral immunity (14). Since antibody molecules themselves have half-lives of only a few weeks, these initial studies indicated that in the absence of antigen-re-exposure it was the cell making the antibody that was long-lived, namely the long-lived plasma cell (LLPC). Subsequent studies have shown that LLPCs primarily live in the bone marrow, but small populations also exist in other tissues. Seminal studies in non-human primates and in normal humans have demonstrated that LLPC could live for decades, up to the lifespan of the individual (15, 16). However, what specifically confers longevity to LLPC (and not, for example to short-lived plasma cell (SLPC)) remains largely undefined.

## 2 Characteristics of LLPCs

As noted above, LLPCs perform a necessary immune function producing antibodies regardless of antigen stimulation, while MBCs require antigen restimulation to produce antibodies. Thus, MBCs can maintain antibody protection against diseases that individual encounters frequently, such as by endemic pathogens. However, pathogens that cause epidemics often essentially disappear from the environment for years or even decades, and generation of neutralizing antibodies by MBCs would require the individual to actually become reinfected, with the associated morbidity and mortality. However, durable antibody titers produced by LLPCs maintain protective immunity against these pathogens that an individual can go years, or even decades, before reencountering (17). As a result of this continuous antibody production PCs have very high metabolic needs and associated stress (18), and it is becoming clear that one of the defining factors that separates LLPCs from SLPCs is the ability of LLPCs to upregulate survival pathways, increase metabolite uptake and handle this metabolic stress (19–21).

Importantly however, LLPCs are not intrinsically long-lived; their lifespan is critically dependent of the microenvironment such that LLPCs removed from their microenvironment die very quickly (22). It is now clear that LLPCs occupy specific LLPC niches in the bone marrow, mucosal tissue and lymphoid tissue, and are dependent on interactions with and signals from these niches for their survival (23); though there is evidence that LLPCs retain some motility (24). The number of LLPC niches appears fixed, which limits number of LLPC (and thus antibody produced) that can exist at a time (17) and results in competition between newly generated LLPC and existing LLPC for niche occupancy (25). Saturation of LLPC niches with aging has been implicated as the reason why aged individuals do not respond to immunization as well as young individuals (26). However, nonmalignant LLPCs do not proliferate and thus occupy additional niche space, which likely decelerates the saturation of the available niches (17, 27).

LLPC longevity is variable, likely reflecting the initial conditions under which the naïve B cells were activated. Amanna et al. demonstrated that human LLPC producing antibodies against the measles virus have an estimated half-life of 3,014 years, while LLPC producing antibodies against recombinant tetanus antigen have a measly half-life of eleven years (15). The antibody titers for different diseases/vaccines followed the same pattern in young and aged individuals, suggesting that though the LLPC niches saturate with age the initial dynamics of the immune response still greatly impact LLPC longevity (15). Understanding what specific mechanisms dictate LLPC differentiation and longevity is essential for developing better vaccines against modern-day pandemics—which is highlighted by the fact that the current mRNA vaccines against SARS-CoV-2 have failed to produce enduring antibody responses from LLPC (28). Conversely given LLPC’s longevity, autoimmune conditions mediated by LLPC are difficult to treat. Furthermore, malignant LLPCs such as in multiple myeloma (MM) rely on many of the same survival pathways as LLPCs (29), and better understanding LLPC survival will also aid in developing treatments for LLPC malignancies.

## 3 LLPC differentiation

LLPC lineage differentiation is thought to begin when naïve B cells are exposed to antigen at the T cell: B cell border in the lymph nodes and other secondary lymphoid organs (30). These activated B cells then infiltrate the germinal center and proliferate rapidly, undergoing somatic hypermutation and affinity maturation (30), and differentiate into MBCs, SLPCs and LLPCs. It has been theorized that the cells producing the highest affinity antibodies were driven to become LLPCs, while those with lower affinity antibodies differentiated into MBCs and SLPCs (31, 32). However, single cell sequencing has shown that LLPCs produce antibodies with a wide array of antigen affinities and differentiate in a much wider array of conditions than previously believed (33, 34). Thus, the mechanisms that drive naïve B cells differentiation into SLPCs, MBCs or LLPCs are still not fully understood. MBCs appear to develop earlier in the germinal center reaction, within the first two weeks post-immunization, while LLPCs develop later 2–5 weeks post immunization—indicating a time-dependent switch (35). However, others have found that LLPCs can develop early in the germinal center reaction and that they continue to develop at a constant rate post-germinal center activation (36).

Many of the factors that are upregulated/involved in PC development are also important in maintaining their function and survival. As PC develop they upregulate expression of the chemokine receptor CXCR4, which allows them to home to the supportive bone marrow microenvironment (BMME). PCs also upregulate expression of transcription factors Interferon Regulatory Factor 4 (IRF4) and the master regulator of plasma cell differentiation B lymphocyte-induced maturation protein-1 (BLIMP-1), which upregulate programs that support high constitutive antibody production such as upregulated amino acid transport (19, 37), metabolic and mitochondrial capacity ((19, 38)) as well as adaptation to the stresses caused by this production (e.g. unfolded protein response (UPR) (39, 40) and reactive oxygen species generation (41)). However, many factors involved in PC differentiation are essential for both SLPC and LLPC generation, and do not specifically drive lineage commitment to LLPC nor define the basis for LLPC longevity.

## 4 Factors intrinsic to LLPC

### 4.1 Overview

**Figure 1:**
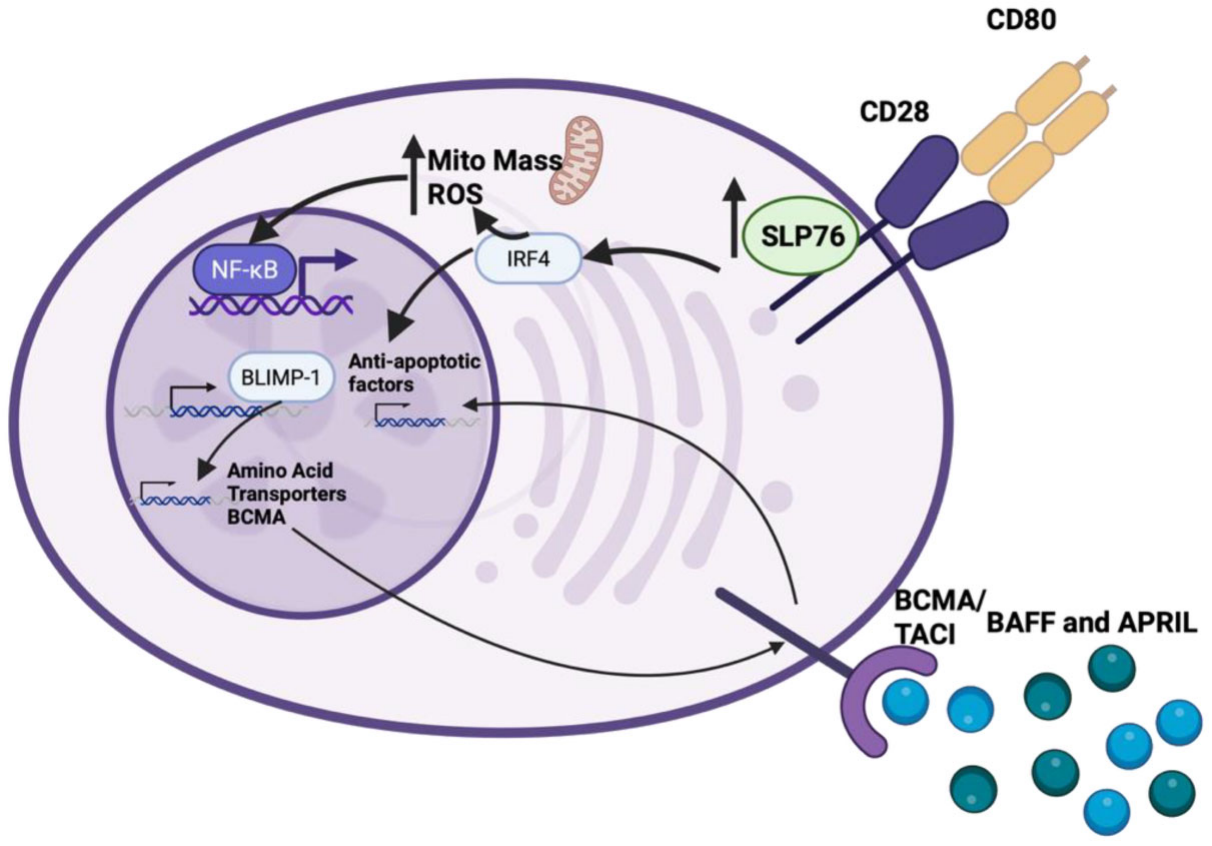
LLPCs rely on a variety of receptor signals, including BCMA, TACI and CD28 to upregulate anti-apoptotic factors and transcription factors important for their survival. Created in BioRender. Reinke, J. (2026) https://BioRender.com/u6s2qy5.

This Section reviews the specific molecular components that are intrinsic to plasma cells that contribute to their survival (**Figure 1**). Cellular metabolism will be reviewed separately in its own section.

LLPC survival requires interaction with extrinsic components of the LLPC niche and the receptors involved in those interactions (as well as their downstream signaling pathways) play a central role in LLPC longevity. It would be predicted that such receptors are expressed only on PC (vs. other B cells), and more specifically LLPC would express both receptors that are essential for all PC survival/function and those uniquely expressed/active on LLPC but not SLPC – allowing only the LLPC to engage/unlock the LLPC niche.

### 4.2 BCMA

B Cell Maturation Antigen (BCMA, *TNFRS17*) is a receptor that was believed to play a vital role in PC survival, though its significance has grown more uncertain over the years (21, 42, 43). BCMA is expressed on both SLPC and LLPC, but not B cells, as its expression is induced by transcription factors BLIMP-1 and IRF4 (44). The ligands for BCMA are the soluble factors BAFF and APRIL, produced by stromal cells, eosinophils and megakaryocytes in the BMME (45–47). O’Connor et al. found that BCMA knockout mice had significantly reduced PCs in the BM, lymph nodes and the spleen, suggesting that BMCA was required for both SLPC and LLPC survival (21). BCMA activation drives the expression of anti-apoptotic protein MCL-1 in PC, providing a possible mechanism for enhanced survival (48). However, BCMA’s role in PC survival has been called into question. In 2025 Menzel et al. demonstrated that BCMA knockout in two different strains of mice had no effect on the number of antigen specific PCs and caused no transcriptomic change in survival signaling up through 126 days post-immunization (43). Due to the lack of transcriptional changes in the BCMA knockout mice, they speculated that BCMA may actually be acting as a sink for BAFF/APRIL and that another receptor for BAFF and APRIL, the Transmembrane Activator and CAML Interactor (TACI), was playing the central role in PC survival (43). However, the authors noted that there are structural differences between mouse and human BCMA, and that BCMA may still be required for human PC survival (43).

### 4.3 TACI

Transmembrane activator and CAML interactor (TACI), like BCMA, is a receptor for soluble factors BAFF and APRIL (49). Tsuji et al. found that in a murine model TACI deficiency impaired BLIMP-1 expression in B cells, which impaired LLPC formation (50). Humans express two splice variants of TACI, the longer isoform is referred to as TACI-L, the shorter isoform TACI-S (51). Garcia-Carmona et al. determined that transfecting TACI-S into human B cells led to increased expression of CD138, BLIMP-1 and XBP1, all associated with plasma cell development, while transfection of TACI-L led to increased expression of B cell marker CD19 (51). TACI-S deficiency impairs BLIMP-1 expression in B cells, which impairs LLPC formation—but TACI is required for the development and survival of both SLPCs and LLPCs, not LLPCs specifically (52). In humans TACI mutations are one of the most common genetic mutations found in patients with common immune variable deficiency, and more specifically IgA deficiency, indicating that TACI also plays an important role in human B cell lineage differentiation (53). Other groups have found that BCMA and TACI both contribute to PC survival and that there may be some redundancy in their functions (42).

### 4.4 CD138

CD138 is a type I transmembrane proteoglycan that can bind to a wide variety of ligands including APRIL and proinflammatory/PC survival cytokine IL-6 (54). High expressing CD138 PCs have increased survival and retention in the BM compared to PCs with low expression (55). Park et al. also demonstrated that high CD138 expression aids PC retention in the BM through binding to fibronectin 1 expressed on stromal cells in the BMME, further aiding in their longevity (55, 56).

### 4.5 CD28

CD28 has been predominantly characterized as a costimulatory receptor on T cells and is activated upon binding to its ligands CD80 or CD86 expressed on antigen presenting cells – in particular dendritic cells (57, 58). In T cells CD28 activation alone has no effect, but in conjunction with T cell receptor (TCR) signaling (“Signal 1”) CD28 costimulation (“Signal 2”) drives full T cell activation that results in proliferation, cytokine production, metabolic adaptation and differentiation (59–61). In mice, CD28 is not expressed on B cells but is expressed on both SLPCs and LLPCs (along with one of its ligands CD86) (62–65). However, in humans CD28 is only expressed on the subset of bone marrow resident PC that have the characteristics of LLPC (66). CD28 expression is inhibited by the master transcriptional regulator of B cell identity PAX5, and the downregulation of PAX5 during B to PC differentiation allows for the upregulation of CD28 expression (67). It was subsequently shown that CD28 activation by itself could not improve the *in vitro* survival of SLPC under stress (serum starvation) conditions but markedly improved the survival of LLPC to the level of serum replete conditions (68). Like T cells, CD28 on LLPC can be activated by binding to CD80 and CD86 expressed on DC, which are a major cellular component of the LLPC BM niche (69, 70).

*In vivo*, Rozanski et al. found that mice with a genetic knockout of CD28 (CD28^-/-^) specifically in the B lineage (T cell expression of CD28 was intact) were able to generate an initial antibody response to immunization equivalent to wild type (WT) mice, but were unable to sustain antigen-specific antibody titers long term and had a significant reduction in antigen-specific LLPC (68). However, loss of CD28 had no effect on the survival of SLPC in the spleen. Consistent with the *in vitro* data, this indicated that CD28 activation was also important for LLPC but not SLPC survival *in vivo*, making CD28 one of the first receptors identified that specifically regulates the survival and longevity of the LLPC subset.

Stimulation of CD28 can produce a variety of signaling cascades. In T cells, activation of CD28 alone does not induce downstream signaling, but in conjunction with TCR/CD3 activation CD28 induces downstream phosphatidylinositol 3-kinase (PI3K), PLCγ and NFκB signals (71). In PC, CD28 activation alone does not transduce any downstream signals in SLPC (similar to T cells) – however, CD28 activation alone on LLPC does activate PI3K, PLCγ and NFκB signaling (41). This explains the pro-survival ability of CD28 specifically in LLPC but not SLPC. The difference between CD28 signaling in SLPC vs. LLPC mechanistically appears to be due to significantly increased SLP-76 expression in LLPCs compared to SLPCs. SLP-76 is an adaptor protein that forms a complex with Grb2/Vav on an intracellular domain of CD28 (41), transducing downstream signaling through NFκB that upregulates expression of the BLIMP-1 (the master regulator of PC identity) and IRF4 transcription factors – resulting in upregulation of multiple pro-survival metabolic and mitochondrial programs in LLPC (described below) (41, 72). It is not currently known what drives higher expression of SLP-76 in LLPC compared to SLPC, but it is tempting to speculate that it is tied to the conditions (for example, high TLR-mediated inflammation (73)) that the B cell is activated in.

In T cells the intracellular domain of CD28 contains two main motifs that bind complexes that connect CD28 receptor activation to downstream signal transduction pathways (71). The Y^170^MNM motif binds the p85 subunit of PI3K and activates downstream PI3K kinase/Akt signaling, and the P^187^YAP^190^ motif binds Grb2/Vav and activates PLCγ—which in turn activates intracellular calcium signaling, protein kinase C activation and NFκB signaling that regulate the AP-1 and NFAT transcription factors. In PC, CD28 activation in LLPC also activates both downstream PI3K and PLCγ/NFκB signaling (72). Functionally, following vaccination mice with knock-in mutations that disable the YMNM motif specifically in the B lineage (CD28-Y170F) had a small reduction in long-term antigen-specific titers but no differences in the number of antigen-specific LLPC vs. WT mice (72). However, vaccinated mice with B lineage disabling mutations in the PYAP motif (CD28-AYAA) had significantly reduced long-term antigen-specific antibody titers as well as antigen-specific LLPC populations. The loss of LLPCs was due to significantly shorter *in situ* survival of antigen-specific LLPC once they were already in the BM (e.g. PC that had been generated and homed to the niche), with a half-life of 27 days for CD28^-/-^ PC and 49 days for CD28-AYAA PC compared to 424 days for WT LLPC population. Unlike LLPC, the number of antigen-specific SLPC remained unaffected by either mutation (72).

### 4.6 BLIMP-1

BLIMP-1 is a master transcriptional regulator in the development of a variety of immune cell types, including plasma cells where it has been characterized as a master regulator of PC identity (74). BLIMP-1 is expressed in SLPCs and LLPCs, but not memory B cells (75). Shapiro-Shelef et al. found that continuous BLIMP-1 expression is required for LLPC maintenance, and global deletion of BLIMP-1 lead to loss of B220^low^CD138^+^ PCs (76). BLIMP-1 supports PC survival in part by upregulating the unfolded protein response (UPR) through regulation of XBP-1 and ATF-6 necessary for PC differentiation (39, 40). However, other studies have shown that BLIMP-1 deletion did not result in the loss of PC but did cause loss of antibody secretion (39). This may be tied to upregulation of expression of amino acid transporters by BLIMP-1; in PCs uptake of amino acids arginine and leucine activate mTORC1, which leads to increased protein and antibody synthesis (39).

### 4.7 IRF4

IRF4 is a critical transcription factor for both SLPC and LLPC differentiation, survival and function. High levels of IRF4 expression at the germinal center stage leads to B cell differentiation into PC and upregulated BLIMP-1 expression (37). PCs require constant IRF4 signaling for survival, as IRF4 loss causes apoptosis although IRF4 does not directly regulate apoptosis (77). Instead, IRF4 regulates genes involved in PC identity and mitochondrial homeostasis (77). At a signaling level, IRF4 regulates expression of SLP-76 that enables CD28 to signal in LLPC, resulting in increased glucose uptake and mitochondrial mass/activity (41). This results in increased oxidative phosphorylation and energy production, and this increased activity results in reactive oxygen species (ROS)-activated signaling, which reinforces LLPC survival (see Metabolism below) (41).

## 5 Extrinsic factors

### 5.1 Overview

**Figure 2:**
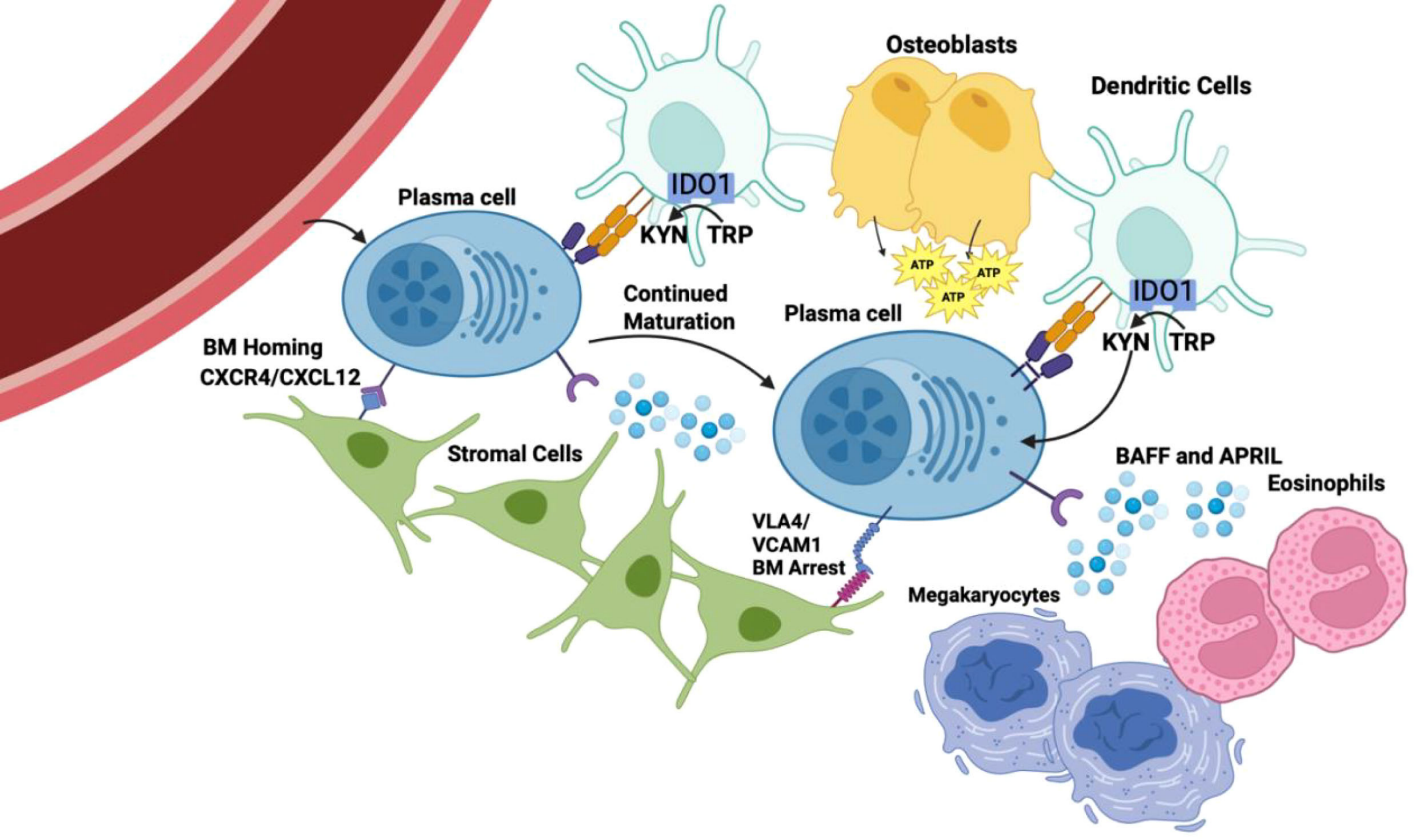
LLPCs are supported by many cell types in the BMME. Created in BioRender. Reinke, J. (2026) https://BioRender.com/hgo0ete.

It is very clear that LLPC are not intrinsically long-lived but are critically dependent on engagement with their microenvironment and the LLPC niche to sustain long term survival and function (**Figure 2**). The availability of a limited number of LLPC niches in turn constrains the size of the total LLPC population – a constraint that is dysregulated in MM, a malignancy of LLPC. Soluble factor such as BMCA/TACI ligands BAFF and APRIL that support LLPC survival are produced by a variety of cells in the BMME (23). Cellular components including stromal cells, dendritic cells, megakaryocytes and eosinophils also have all been shown to support LLPC survival in the bone marrow, although later studies complicated the idea of what constitutes a stable bone marrow LLPC niche and what cells are actually necessary for LLPC survival. .

### 5.2 Motility

Initial studies determined that LLPCs remained sessile once they had translocated to the bone marrow and did not migrate either in the BM or outside of it (78, 79). However, more recent studies found that BM PCs migrate within the bone marrow and occasionally recirculate in the body, indicating that the LLPC niche may be more dynamic than previously described (24). VLA-4 promotes PC arrest in BMME (24), and Koike et al. have shown that LLPCs undergo further differentiation in the BMME and are motile in the early stages but become less motile as they settle and upregulate expression of VLA-4 (80). Therefore VLA-4 expression is likely necessary for LLPC longevity by anchoring them in their permanent niche.

### 5.3 Competition between LLPC for the niche

Durable antibody titers from LLPCs can be generated at any point over a lifespan, indicating that the LLPC niche never becomes fully saturated and/or that newly generated LLPCs have ways of out-competing the old for niche occupancy—voting them off the island as it were. In 2005 Odendahl et al. found in humans that approximately one week after tetanus vaccination, as the tetanus-specific PCs were released from the secondary lymphoid organ to home to their microenvironment, a population of non-tetanus specific IgG+ plasma cells appeared circulating in the blood—suggesting that the newly formed PCs had successfully displaced existing PCs in the niche (25). These findings suggested that the ability of LLPCs to “stick” to the niche is an important component of their longevity. Similarly Slocombe et al. found that the TNF-mediated inflammation caused by an infection lead to a “dramatic mobilization” of BM LLPCs into recirculation, leading to reduction in pre-existing antigen-specific titers that indicated the permanent loss of some PCs (81)—though not all recirculating PC necessarily die (24). Mohr et al. postulated that the greater the number of new PCs generated during an infection, more preexisting BM PCs are displaced from their niche and a greater proportion of new PCs can home to the niche—suggesting the severity of the initial immune response is important for “clearing out space” to make room for the newly generated LLPCs (82). However, Robinson et al. argue that LLPC lifespan is not determine by niche competition, but instead by an intrinsic transcription program (83). They found that when production of new PCs was blocked the rate of PC loss in the BM and spleen was not significantly different than when there was competition with new PCs; they instead found that a specific transcriptional program driving high CD138 and CD93 expression was associated with true longevity (83). In a hybrid model of niche competition vs. LLPC-intrinsic programs, Simons and Karin developed a “self-tuning” mathematical model for LLPC longevity based on vaccine responses to hepatitis A, B and HPV, that posits that the competition over survival factors in the niche has an effect on the PC’s gene regulatory network, subtly pushing the PC to either longevity or removal from the niche, with factors such as high CXCR4 expression skewing towards longevity (84).

### 5.4 Mesenchymal stromal cells

Mesenchymal BM stromal cells (BM SC) support LLPC homing to and adhesion with the BMME necessary for LLPC survival and sustained function. As LLPCs differentiate they begin to express CXCR4 (the ligand for the CXCL12 chemokine) and the VLA-4 adhesion molecule (85). CXCR4 blockade reduced the number of antibody-secreting cells in the bone marrow by 60% (86), demonstrating its importance in PC homing to the BMME. BM SC also express the ligands for many of the adhesion molecules expressed by BM PCs; VCAM-1 (for VLA-4), ICAM-1, SPP1, HAS2, CXCL12 (45). Interestingly however, while knocking out VLA-4 in PC led to a significant decrease in IgG secretion, knocking out VCAM-1 on BC SC had no impact – suggesting that PCs may not be fully reliant on BM SC expression of VCAM-1 for bone marrow arrest (87).

BM SC also support PC survival and function. Co-culturing BM PC with BM SC *in vitro* extends the lifespan and IgG production of a subpopulation of these PC from 7 to 28 days and knocking out IL-6 in the BM SC significantly decreased IgG production (87). BM SC also produce low levels of BCMA ligands BAFF and APRIL (45). In addition to soluble factors, PC survival is dependent on direct contact with BM SC, as culturing BM PCs in direct contact with BM SC results in significantly increased survival and antibody production vs. BM PC separated from BM SC in a transwell co-culture system (45). Many of the adhesion molecules expressed by BM SC can trigger PI3K and NFκB signaling in the adhering cell (45), and contact with stromal cells has been shown to inhibit apoptosis through inhibition of caspase 3 through PI3K signaling and through inhibition of caspase 12 through NFκB signaling (20).

### 5.5 Osteoblasts

Osteoblasts are also derived from mesenchymal stem cells and have been shown to support LLPC survival in the BMME through the release of extracellular ATP through PANX3 (88). LLPCs sense and take up the extracellular ATP though P2RX4, and in murine models of antibody-mediated autoimmune kidney disease blockade of P2RX4 leads to loss of BM PC and a decrease in auto-antibody production (88). Interestingly however, MM causes osteoblast apoptosis and suppresses osteoblast activation through secretion of soluble WNT inhibitors, leading to osteolytic lesions (89, 90) – suggesting that at least in the malignant setting, osteoblasts are not essential for MM cell survival.

### 5.6 Megakaryocytes and eosinophils

In addition to mesenchymal cells, cells derived from different hematopoietic lineages have been reported to be essential components of the supportive microenvironment for BM PC. Megakaryocytes and eosinophils both produce soluble factors (IL-6, BAFF and APRIL) that support PC survival in the bone marrow (46). Mice with defects in megakaryocyte formation have impaired accumulation of antigen-specific BM PC (46). Similarly, Chu et al. found that eosinophil-deficient mice had severely reduced numbers of BM PC, and that selective depletion of eosinophils led to BM PC apoptosis *in vivo* (91). However, later studies have shown that mice genetically deficient in eosinophils or wild-type mice depleted of eosinophils do not have loss of BM PC (total or antigen specific) (92, 93) – and the difference attributed other cell types producing the same survival factors as eosinophils whose presence in the BMME is influenced by murine genetic strain and differences in microbiota (92, 93).

### 5.7 Dendritic cells

Given the importance of CD28 activation specifically for LLPC survival, it would be anticipated that cells expressing the CD28 ligands CD80 and CD86 would be key cellular components of the LLPC niche. CD80/CD86^+^ cells are predominantly immune antigen-presenting cells (APC) that include B cells, monocyte/macrophages and DC (myeloid/conventional and plasmacytoid). DC are best characterized as the primary professional APC that drive T cell activation (94), but they also support normal PC differentiation (95–97) via cell-cell contact and production of the pro-PC survival cytokines IL-6 and APRIL/BAFF (98). This is supported by the demonstration that conventional dendritic cells (cDC) support long-term LLPC survival and Ig production *in vitro* in a CD28-dependent fashion, and BM CD80^+^ DC are in direct contact with LLPC *in vivo (*68), as well as other elegant studies showing both DC and regulatory T cells (Treg, expressing CTLA4, a member of the CD28 superfamily that also binds CD80/CD86) are essential LLPC niche components and are in direct contact with the BM PC (99). Specific depletion of DC *in vivo* (in particular, DC expressing indolamine 2, 3 dioxygenase (IDO1, below)) causes loss of long-lived antigen-specific antibody titers as well as loss of antigen-specific LLPC in the bone marrow (100). Mirroring normal LLPC biology, we and others have found that in MM that cDC, plasmacytoid dendritic cells (pDC) and macrophages are preferentially recruited into myelomatous regions in patient BM and protect MM against chemotherapy-induced death (101–106), and that pDC (106) and cDC (101, 107–109) protect in a cell-contact dependent manner that requires MM CD28 (64, 101, 109).

In addition to CD28 engagement to DC CD80/CD86 signaling LLPC, it also modulates the LLPC ME by triggering “backsignaling” through CD80/CD86 to the DC (110). This induces DC production of IL-6 and the immunosuppressive enzyme indoleamine 2,3 dioxygenase 1 (IDO1) (109–112), which has been previously shown in T:DC interactions (57). IL-6 is a well-characterized PC survival cytokine that protects PC survival in combination with other soluble factors such as BAFF and APRIL (42, 113). Interestingly, co-culturing BM PC with IL6^-/-^ DCs significantly reduced IgG production but did not affect overall PC survival (68) – suggesting that in the context of LLPC: DC direct interaction, IL-6 is dispensable for PC survival. IDO1 catabolizes the essential amino acid tryptophan (Trp) and depletes it from the microenvironment—resulting in the generation of Treg (114) as well as suppression of effector T cell activation through Trp-depletion-mediated integrated stress response (115–117). IDO1 was initially described as essential for physiological inhibition of allo-reactive maternal T cells at the maternal-fetal placental interface (118), and has subsequently been found to play a significant role in generating physiological immune-privileged sites in the brain, eye and testes (119–122). Thus, in addition to directly transducing a pro-survival signal to the LLPC, CD28 engagement of DC CD80/CD86 elicits a stromal DC-produced soluble ME that supports LLPC survival and generates a local immune-privileged site that prevents potential “anti-self” immune responses against the neoantigen idiotype of the antibody the LLPC is producing. This may be particularly important given that niche DC can take up, process and present this neoantigen to T cells, that have not been thymically tolerized, to new self-antigens like antibody idiotypes. From an evolutionary standpoint, preventing T cell-mediated killing of LLPC that are producing antibodies preventing reinfection by a deadly pathogen would be an important advantage.

In addition, IDO1 catalyzes the rate limiting step in the catabolism of Trp to the downstream metabolite kynurenine (Kyn), which is the endogenous ligand of the aryl-hydrocarbon receptor (AHR) (100). In the B lineage AHR is most highly expressed in PC (123) and an AHR-regulated gene signature unique to LLPC has been identified (124). Lightman et al. found that co-culturing DC with BM PC *in vitro* leads to upregulation of IDO1 activity in DC that supports LLPC survival and function (IgG production) in a CD28/CD80-CD86 and AHR dependent manner (125). Interestingly, Kyn upregulates LLPC CD28 expression – suggesting a positive reinforcing feedback loop where the CD28 - CD80/CD86 interactions between LLPCs and DCs upregulates IDO1 expression➔IDO1 catabolizes Trp to Kyn➔Kyn activates AHR in the LLPC➔the LLPC upregulates CD28 expression. *In vivo*, this group found that selective depletion of IDO1-producing DC (or pharmacological inhibition of IDO1) led to loss of long-term antigen-specific antibody titers and significantly reduced numbers of antigen-specific LLPC in the bone marrow, while SLPCs were unaffected (125). Similar to LLPC, MM cells also induce DC to produce IL-6 and IDO1 to create a pro-survival and immunosuppressive microenvironment (109) and are dependent on AHR activation for their survival (126).

### 5.8 Spleen

The red pulp of the spleen contains CXCL12 expressing stromal cells, similar to the BMME, allowing the PCs to home to the splenic microenvironment through a similar mechanism, and PCs sometimes recirculate from the BMME to the spleen (24, 127). Though the spleen is typically characterized as the residence for SLPCs, several populations of LLPCs reside here, including IgM and IgE secreting LLPCs (128, 129). Splenic LLPCs rely on many of the same survival signals as their BMME counterparts—for example, splenic stromal cells produce the pro-survival cytokine IL-6 (127). Thai et al. found a population of BAFF-producing neutrophils in the splenic PC environment that also support PC survival (130). They also found splenic PCs in close contact with CD4 T cells, and while CD4 depletion alone did not affect PC survival, CD4 depletion in combination with the stress of CD20 depleting therapy did—indicating that CD4 T cells may support splenic PC survival under stress conditions (130). Jang et al. found a population of spleen-derived myeloid cells in a murine lupus model that had a more inflammatory phenotype than those in the BMME, which skewed CD4 T cells in the spleen to an inflammatory phenotype and increased numbers of splenic LLPCs, indicating the formation of an inflammatory feedback loop that contributed to pathology (131).

### 5.9 Lamina propria

There is a measurable clonal overlap between IgA^+^ LLPCs found in the lamina propria and IgA^+^ LLPCs found in the BMME, indicating they share a common origin, though they do not recirculate between tissues the way PCs in the spleen and BMME do (132). Unlike the BMME and splenic LLPCs which rely largely on the CXCR4/CXCL12 interaction to home to the microenvironment, B cells and PCs that migrate to the mucosal tissues rely on expression of integrin α4β7, which binds to MadCAM-1, expressed on intestinal endothelial cells, as well as other chemokines and chemokine receptors for more specific tissues, such as CCR9/CCL25 for the gut and CCR10/CCL28 for the lactating mammary gland (133–136). Intestinal epithelial cells also express BAFF and APRIL, supporting LLPC survival in lieu of stromal cells (133). Intestinal LLPCs can form directly in the Peyer’s patches of the lamina propria, through interactions with antigen-presenting DCs and CD4 T cells (133).

Intestinal LLPC formation is more directly linked to diet/metabolism compared to their BMME and splenic counterparts. Diets high in cholesterol cause the production of 25-hydroxycholesterol (25-HC) in DCs in the intestinal microenvironment, which restricted the activation of transcription factor SREBP2; blocking cholesterol metabolism to 25-HC and significantly increased intestinal IgA secretion (137). Intestinal LLPCs do not express CD28, and thus do not induce the production of AHR through CD28/CD86 interactions with DCs in the intestinal microenvironment (138, 139), However, intestinal LLPCs are exposed to AHR ligands through diet and through the indole metabolites produced through the microbiome, and still undergo activation of AHR-mediated survival pathways (133, 140–142). Mucosal LLPCs produce lower amounts of antibody than their counterparts in the BMME and spleen, indicating further metabolic differences that will be discussed below (143).

## 6 Metabolism

### 6.1 Overview

**Figure 3:**
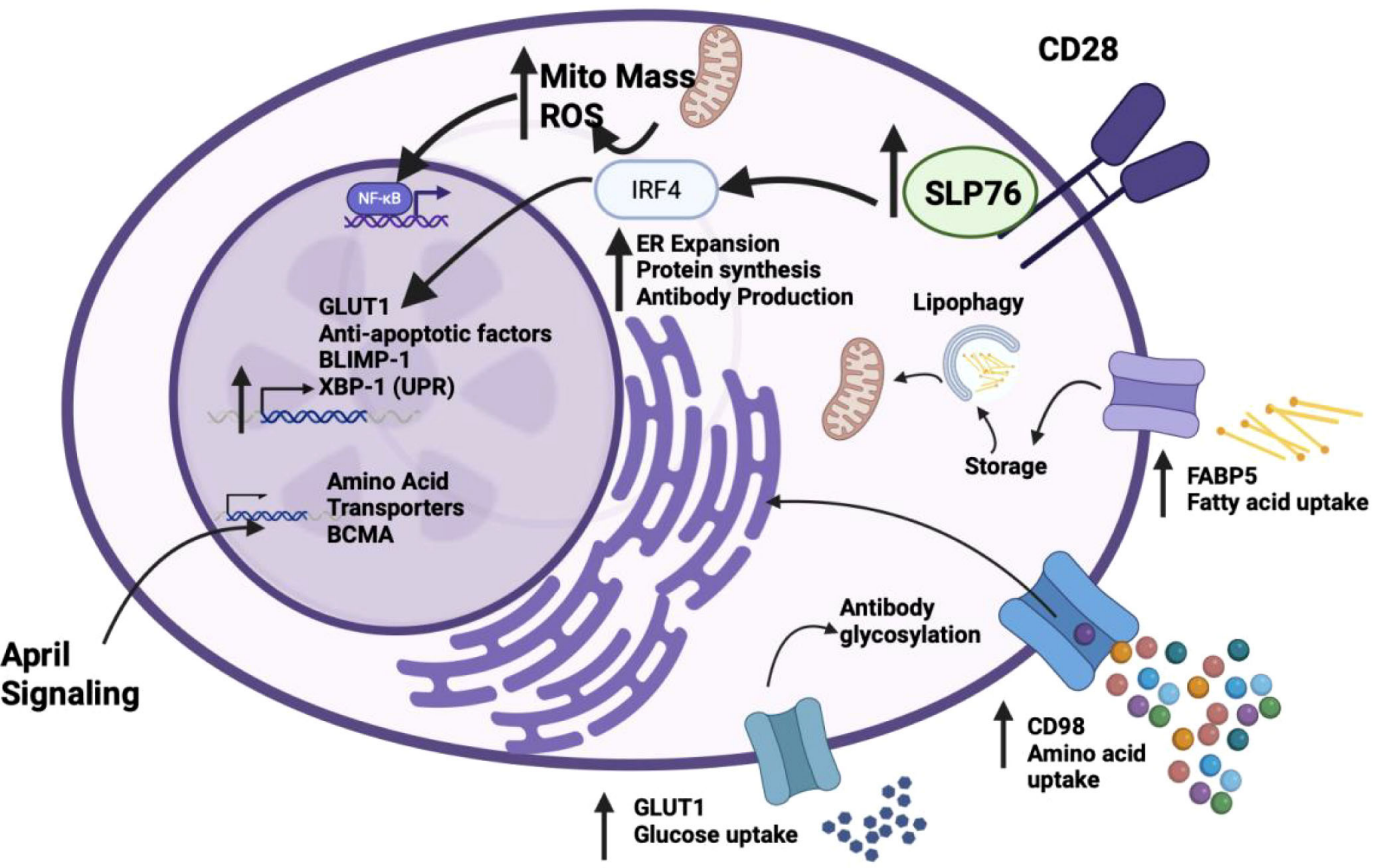
LLPCs rely on upregulation of many metabolic pathways for survival. Created in BioRender. Reinke, J. (2026) https://BioRender.com/dlxfc92.

Because of the high biosynthetic activity and associated energy demands that PC require for continual production of antibodies, emerging data now points to metabolic adaptation as essential for PC survival in general and for the longevity of LLPCs (vs. SLPCs) in particular (19). These adaptations include enhanced uptake of a diverse group of metabolic substrates (including glucose, lipids and amino acids), increased protein biosynthetic capacity and upregulation of sustained energy production (**Figure 3**).

### 6.2 Metabolite uptake

Glucose plays a central role in both biosynthesis and energy production in all cells, and it has been shown that increased glucose metabolism is an important component in PC differentiation such that blocking glucose metabolism decreases differentiation to CD138^+^ PC (144). LLPCs take up more glucose compared to SLPCs (19), consistent with the finding that CD28 activation upregulates expression the GLUT1 glucose transporter and glucose uptake in LLPC (but not SLPC) (41). Interestingly, one carbon tracking studies demonstrate that LLPC primarily utilize glucose for antibody glycosylation rather than energy production, although loss of the ability to use glucose-derived pyruvate for energy production causes selective loss of LLPC (38, 145). Studies in both normal LLPC and their transformed MM counterparts demonstrate that they are largely reliant on long chain fatty acid (LCFA) oxidation as their primary energy source, which generates more ATP per fuel molecule (38, 41, 146). This dependency may be greater in MM cells since they are proliferating and is consistent with the observations that adipocytes are part of the supportive MM microenvironment and that both fatty acid transporters/binding proteins (especially FABP5) and autophagy/lipophagy are essential for MM cell survival (146, 147). In addition to glucose and lipids, to meet the demand for antibody production LLPCs increase their amino acid uptake (145), and LLPCs have higher expression of the amino acid transporter CD98, which is regulated by BLIMP-1, compared to SLPCs (19, 39). Of note, AHR is involved in the regulation of many transporters and enzymes involved in the processing and uptake of small molecules and metabolites. AHR has also been implicated in altering fatty acid metabolism (148) – although whether it plays this role in LLPC is unknown.

### 6.3 Endoplasmic reticulum

PCs undergo an expansion of the endoplasmic reticulum (ER) to allow for increased antibody production, requiring lipids and cholesterol to support the expanded ER membranes (149). Increased protein synthesis increases ER stress, and SLPCs are more susceptible to apoptosis induced through ER stress through the ER-associated caspase 12 compared to LLPCs (150). The microenvironment plays a large role in allowing LLPCs to overcome their ER-induced apoptotic stress, as stromal cell-produced APRIL induces NFκB signaling and prevents caspase 12 activation (20). LLPCs are also able to better handle ER stress through upregulation of XBP-1, a transcription factor that helps handle the UPR, and downregulation of XBP-1 repressor PAX-5 through expression of BLIMP-1 (151, 152).

### 6.4 Mitochondria

Due to the increased energy demands needed to support PC biosynthesis, PC differentiation and survival is dependent on increased levels of mitochondrial function and a metabolic switch from glycolysis to oxidative phosphorylation of LCFA (38, 41, 146). Urbanczyk et al. created a cell-specific reduction of mitochondrial DNA (mtDNA) in developing B cells, using a CD23cre locked dominant negative mutation of the mitochondrial helicase Twinkle. Reduction in mtDNA dampened oxidative phosphorylation, but increased glycolysis (153). The reduction in mtDNA altered metabolism and restricted PC differentiation, dampening expression of BLIMP-1 and mTORC1, but did not affect B cell differentiation or total numbers (153).

LLPCs in the BM have increased mitochondrial mass compared to SLPCs found in the spleen, indicating that increased mass and mitochondrial function may be necessary to meet the high energy demands PCs have long-term (38). This difference is in part due to activation of LLPC CD28 upon binding to niche DC CD80/CD86, which results in rapid upregulation of both mitochondrial mass and function (as measured by mitochondrial membrane potential)—which is not seen with CD28 activation in SLPC (41). The increased mitochondrial membrane potential is due to increased basal and maximal mitochondrial respiration, reflecting increased oxidative respiratory capacity of LLPC mitochondria. Interestingly, the increased reactive oxygen species (ROS) generated by increased mitochondrial activity activates pro-survival NFκB signaling – directly linking increased energy generation/mitochondrial capacity to LLPC survival and longevity (41).

Like normal LLPCs, MM cells express both CD28 and CD86, and high expression of CD28 and CD86 is associated with disease progression and poor survival (64). The molecular signaling underlying CD28-mediated effects in MM cells mirrors many aspects of CD28 signaling in LLPC and T-cell biology. In both, CD28 activation rapidly triggers the phosphorylation of the p85 subunit of PI3K, which initiates a cascade that culminates in Akt activation and subsequent downstream effector activity (101, 108). CD28-mediated PI3K/Akt activation leads to NFκB nuclear translocation through IκB degradation, upregulating expression of IRF4 (41). IRF4 acts as a critical metabolic regulator of MYC expression, which in turn controls mitochondrial biogenesis through peroxisome proliferator-activated receptor gamma coactivator 1-beta (PGC-1β) (154). This signaling hierarchy establishes a direct link between CD28 co-stimulation and mitochondrial metabolic capacity. Knockdown of CD28 has been shown in human MM cell lines to significantly reduce the expression of mitochondrial membrane proteins such as VDAC1, TOMM20, and SUCLA2, which are crucial for mitochondrial architecture and function, likely regulated through PGC-1β. Consistent with this, comparative analysis of CD28-high versus CD28-low MM populations reveals a 30-50% increase in mitochondrial mass in the former (41). This increase correlated directly with an increase in respiratory capacity with CD28-high cells, demonstrating 1.5-fold higher basal oxygen consumption and 2-fold higher maximal respiratory capacity rates.

In addition to “more” mitochondria, studies in MM suggest that MM/LLPC mitochondria are also “better” and can run at higher metabolic levels because of upregulated homeostatic mechanisms that increase baseline tolerances to allow for this. One example of this was reported by Qin et al. who have found that MM cells upregulate the mitochondrial endoprotease CLP, which is a critical component of the efflux arm of mitochondrial protein quality control that tightly regulate mitochondrial enzyme pools (155, 156). CLP inhibition disrupted mitochondrial metabolism, ATP production and MM cell death, further strengthening the connection between increased mitochondrial capacity and LLPC survival and longevity.

However, the LLPC dependance on increased mitochondrial capacity and oxidative phosphorylation may not be universal and may be tissue specific. IgA+ LLPCs in the gut lamina propria have significantly decreased mitochondrial mass compared to IgA-producing LLPCs in the BMME (143). Gut lamina propria IgA+ LLPCs also remain largely dependent on glycolysis, unlike the BMME LLPC’s reliance on oxidative phosphorylation and autophagy (143). This difference in metabolism may be due to differing metabolic needs, as gut IgA+ LLPCs produce significantly fewer antibodies than BMME IgA+ LLPCs – potentially using less for antibody glycosylation and more for energy production (143).

### 6.5 Autophagy and lipophagy

In addition to exogenous sources, are there other sources of fuel that can sustain LLPC metabolism? This may be particularly important during times of bone marrow stress (e.g. emergency granulopoiesis with sepsis) when exogenous sources can be depleted. Intracellularly, LLPC are rich in lipid membranes (ER, Golgi) and lipid stores (in the form of droplets), which can be converted into fuel molecules and other metabolic substrates by autophagy. Autophagy is a process of cellular recycling in which a cell can degrade intracellular components to metabolites, through formation of autophagosomes that deliver components to the lysosome for degradation (157). Autophagy is vital for PC development (158). Pengo et al. found that knockout of Autophagy-Related Gene 5 (ATG5) led to increased ER stress and greater PC death, resulting in decreased numbers of antigen-specific PCs in the BM (159). Another study also found that deletion of ATG5 in B cells decreased the production of IgG and IgM in the BM and gut-associated immune compartments, though not in the spleen indicating ATG5 was less necessary for SLPC survival (160). ATG5 deletion also led to an inability to upregulate BLIMP-1 and XPB1 necessary for PC differentiation, indicating that autophagy plays an important role in the PC developmental programs (160).

Lipophagy is a form of autophagy in which intracellular lipids stored in lipid droplets are taken up by the autophagosome and broken down by the lysosome, resulting in free LCFA (161, 162). This can be used to fuel mitochondrial beta-oxidation, allowing PCs to meet the high energy demand (38, 159, 161). As PC mature, they begin to rely on lipophagy and fatty acids as a primary energy source. Blocking fatty acid oxidation reduced CD138^+^ PC differentiation (144, 163). Mirroring this, MM cells are also dependent on beta-oxidation of long-chain fatty acids and pro-survival signals like CD28 activation-induced lipophagy + increased LCFA-fueled oxidative phosphorylation that results in increased survival and treatment resistance (146). Thus, lipid metabolism represents a novel treatment target for MM (164).

## 7 LLPCs and disease

### 7.1 Autoimmunity

LLPCs are involved in the pathology of a variety of antibody-mediated autoimmune diseases including systemic lupus erythematosus, Sjogren’s disease and a number of neurological disorders (165, 166). LLPCs are also found in nasal polyps and may be involved in their prolonged survival (167). Although not truly pathology, LLPC are the primary source of long-term alloantibodies (e.g. induced by blood transfusions, etc.) that are the primary cause of host antibody-mediated graft rejection in solid organ transplantation (168, 169).

Given LLPCs have multiple factors that support their survival, both intrinsic and in their microenvironments, when they cause pathology, they can be very difficult to eliminate. LLPCs do not express CD19 or CD20 and therefore are resistant to many of the B cell depleting therapies used to treat autoimmune diseases (169). In fact, Mahévas et al. showed in murine models that some CD20 depleting therapies alter the function of the spleen and promote the differentiation of autoreactive SLPCs into autoreactive LLPCs, a possible contribution to treatment failure (170). Thai et al. found that using an anti-BAFF or anti-CD4 antibody in combination with a CD20 depleting therapy significantly reduced the number of splenic LLPCs, indicating that by targeting the PC survival pathways they could mitigate the autoreactive skewing caused by anti-CD20 therapy alone (130). The success of BCMA-targeting chimeric antigen receptor (CAR) T cells in eradicating MM cells and normal PC in MM patients has also led to the development of BCMA and CD19-targeting CAR T cells to target both the LLPC and B cell compartments in otherwise refractory rheumatologic diseases (171, 172).

### 7.2 Multiple myeloma

MM is a hematological malignancy caused by abnormally proliferating malignantly transformed LLPC. It is currently considered incurable and has a five-year survival rate of 62% (SEER). Although malignant, MM retains many of the biological characteristics of LLPC—which is underscored by the fact that the majority of treatments that have been developed for MM target aspects of normal LLPC biology (29, 173).

As the precursor stages of MM, Monoclonal Gammopathy of Uncertain Significance (MGUS) and Smoldering Multiple Myeloma (SMM), develop there is an increase in the level of soluble BCMA in serum, indicating an increase in BCMA expression (174, 175). In individuals with MGUS and SMM, higher levels of soluble BCMA are associated with progression to MM (175). These findings suggest that BCMA plays an essential role in MM progression and survival, which has been the central rational for targeting BCMA through chimeric antigen receptor (CAR) T cells and bispecific antibody T cell engagers (BiTEs) that have shown remarkable clinical efficacy (176, 177). However, antigen escape via BCMA deletion or mutation has been demonstrated to be a major mechanism for acquired MM resistance (in particular for the BiTEs) (178), and dual targeting BCMA and TACI has been proposed as a strategy to overcome single antigen escape in MM (179, 180).

Given its importance in MM, to directly target CD28 activation we have found in preclinical models that CTLA4-Ig (abatacept), a chimeric CTLA4 -Ig fusion protein that binds to CD80/CD86 and blocks CD28 activation, sensitizes MM cells *in vitro* and *in vivo* to death signals, including chemotherapy (108). Abatacept is FDA-approved for the treatment of rheumatoid arthritis, and we have recently completed a single-arm, open-label, multi-center study phase II trial targeting CD28 in MM with abatacept + ixazomib + dexamethasone to overcome resistance to chemotherapy (NCT03457142). Briefly, in this trial we tested the efficacy and safety of combining abatacept with the oral proteasome inhibitor (PI) ixazomib (ixa) + dexamethasone in relapsed/refractory (RR) MM patients who had relapsed after treatment with the PI bortezomib (181). Previous studies have shown that only 11% of patients with prior bortezomib expose respond to ixa + dex alone (11% clinical benefit rate, CR+PR+SD), with event free survival of 5.7 months (182), most likely due to acquired resistance to proteasome inhibitors. We found that the addition of abatacept compare favorably to previous findings of ixa + dex alone for overall response rates (35.7% abatacept + ixa + dex vs. 11% ixa + dex alone), clinical benefit rate (93% vs. 11%), and PFS (11.3 months vs. 5.7 months (event free survival)). These findings suggest that blockade of CD28 activation on MM cells with abatacept can reverse acquired treatment resistance in MM, which is ultimately why the vast majority of MM patient succumb to their disease.

## 8 Discussion

Long-lived plasma cells and their continual antibody production are essential for durable long-term immunity that protects against the morbidity/mortality of infectious diseases—particularly those involving pathogens that cause epidemics but are only sporadically present in a population. From a whole organism standpoint, having a population of highly metabolic long-lived cells that can be repeatedly expanded with every infection presents its own space and resource management challenges. Thus, both the survival and regulation of LLPC populations are a tightly woven interplay of PC intrinsic and extrinsic factors.

Why are long-lived plasma cells long lived, and what confers that longevity that distinguishes LLPC from short-lived plasma cells? Some of the underlying mechanisms have been described in this review, but the majority remain largely undefined. What leads to LLPC differentiation from naïve B cells is still not fully clear, as LLPCs can develop from both germinal center-dependent and GC-independent reactions with IL-21, CD40 and BCR signaling all playing an important role in LLPC development (34, 183–186) – although it is not clear if these factors distinguish LLPC vs. SLPC lineage commitment. The ability of CD28 to transduce a pro-survival signal in LLPC while it does not in SLPC clearly distinguishes the two PC subsets, and it is attractive to speculate that factors in B cell activation/PC differentiation that regulate expression of SLP-76 (which enables CD28 signaling) play a significant role in determining whether a B cell is going to become a LLPC.

One of the first clearly distinguishing features of LLPC vs. SLPC is where they live. LLPC have been classically described to be in the bone marrow while SLPC are found in secondary lymphoid organs like the spleen, although that distinction is an oversimplification. However, given that LLPC survival is completely dependent on specific interactions with the microenvironment, this suggests there is a specific LLPC pro-survival niche that any newly minted LLPC needs to be able to find and unlock. LLPC expression of CXCL12 and the VLA-4 appear to allow them to uniquely home to and stick to the LLPC niche, although later observations that LLPCs are motile in the BMME as they continue to differentiate suggest that the LLPC niches are more dynamic than previously believed (24).

Once in the niche, LLPC need to unlock the pro-survival components through specific “key” interactions. Cells like megakaryocytes and eosinophils secrete pro-survival BCMA ligands BAFF and APRIL to support LLPC survival, though how essential each specific cell type is for LLPC survival remains debated (46, 92). However, unlocking of pro-survival niche appears in part to involve activation of LLPC CD28 to transduce pro-survival signals (which does not happen in SLPC), which happens upon engagement of CD80/CD86 on DC in the LLPC niche. Furthermore, this interaction back-signals to the niche DC to upregulate expression of IL-6 (pro-survival/function) as well as IDO1. IDO1 likely plays at least 2 central roles in supporting LLPC longevity through the establishment of an immune-privileged site as well as generation of AHR ligand Kyn that upregulates LLPC CD28 expression – which is niche feedback that reinforces that the LLPC has successfully engage the LLPC niche.

Finally, it is quite striking that LLPC and SLPC are transcriptionally similar but are metabolically very different (19), supporting the biological framework that the central nexus of LLPC longevity involves metabolic adaptation that increases the capability for sustain high level biosynthesis in terms of both resources needed (e.g. energy production) as well as mitigation of the associated stresses (production of ROS from increased oxidative phosphorylation, induction of unfolded protein responses, etc.). It is clear that LLPCs undergo many metabolic changes as they differentiate and engage the LLPC niche – including increasing mitochondrial mass/function, glucose and amino acid uptake and fatty acid oxidation (38, 41, 145). This increased capacity appears to be intrinsically linked to the ability of the PC to engage and unlock the pro-survival components the LLPC niche, which dovetails with the regulation of the overall LLPC population through niche availability.

In human health, understanding what drives PC longevity and generation of durable humoral immune responses will be essential for rational development of effective vaccines, especially novel formulations like mRNA vaccines that differ substantially from “traditional” attenuated whole pathogen or recombinant protein formulations. Perhaps the greatest impact will be in the development of new treatments against antibody-mediated autoimmune diseases and PC malignancies like MM, as it is increasingly clear that MM is still very dependent on many of the survival pathways of their normal LLPCs counterparts (29, 173). Given many of these pathways have not yet been therapeutically targeted in MM, an understanding of why long-lived plasma cells are long-lived will point to novel therapeutic approaches for this incurable malignancy.
